# Medication exposure during pregnancy: a pilot pharmacovigilance system using health and demographic surveillance platform

**DOI:** 10.1186/1471-2393-14-322

**Published:** 2014-09-15

**Authors:** Dominic Mosha, Festo Mazuguni, Sigilbert Mrema, Salim Abdulla, Blaise Genton

**Affiliations:** Ifakara Health Institute, Rufiji, HDSS, P.O Box 40, Rufiji, Tanzania; Swiss Tropical and Public Health Institute, University of Basel, Basel, Switzerland; Department of Ambulatory Care and Community Medicine & Division of Infectious Diseases, University Hospital, Lausanne, Switzerland

**Keywords:** Medication, Pregnancy, Pharmacovigilance

## Abstract

**Background:**

There is limited safety information on most drugs used during pregnancy. This is especially true for medication against tropical diseases because pharmacovigilance systems are not much developed in these settings. The aim of the present study was to demonstrate feasibility of using Health and Demographic Surveillance System (HDSS) as a platform to monitor drug safety in pregnancy.

**Methods:**

Pregnant women with gestational age below 20 weeks were recruited from Reproductive and Child Health (RCH) clinics or from monthly house visits carried out for the HDSS. A structured questionnaire was used to interview pregnant women. Participants were followed on monthly basis to record any new drug used as well as pregnancy outcome.

**Results:**

1089 pregnant women were recruited; 994 (91.3%) completed the follow-up until delivery. 98% women reported to have taken at least one medication during pregnancy, mainly those used in antenatal programmes. Other most reported drugs were analgesics (24%), antibiotics (17%), and antimalarial (15%), excluding IPTp. Artemether-lumefantrine (AL) was the most used antimalarial for treating illness by nearly 3/4 compared to other groups of malaria drugs. Overall, antimalarial and antibiotic exposures in pregnancy were not significantly associated with adverse pregnancy outcome. Iron and folic acid supplementation were associated with decreased risk of miscarriage/stillbirth (OR 0.1; 0.08 – 0.3).

**Conclusion:**

Almost all women were exposed to medication during pregnancy. Exposure to iron and folic acid had a beneficial effect on pregnancy outcome. HDSS proved to be a useful platform to establish a reliable pharmacovigilance system in resource-limited countries. Widening drug safety information is essential to facilitate evidence based risk-benefit decision for treatment during pregnancy, a major challenge with newly marketed medicines.

## Background

Access to different therapeutic drugs such as antibiotics, antimalarial and antiretroviral (ARVs) have improved in recent years in most African countries, including Tanzania, thanks to the efforts facilitated by government, private sector and donor agencies [1, 2]. Safety of some of these therapies is unknown during pregnancy because pregnant women are not involved in clinical trials during the drug development process and hence, most pharmaceutical products come to market with little human data available regarding safety in pregnancy. Studies from animal models have been used to provide safety information during pregnancy at the time the new drug is approved. However, such findings are not easily translated into human risk. In most cases, information regarding safety of product or drug use during pregnancy is collected post product approval [3, 4]. Sufficient and valid data on safety of drug use during pregnancy is of high public health importance so as to facilitate evidence based risk-benefit decision among health providers.

Drug exposure during pregnancy in Western Europe and US is reported to have increased in the past 10 years [5, 6]. In most developing countries, where proper drug monitoring system during pregnancy does not exist, it is difficult to know the magnitude of drug exposure in pregnancy. There are few studies in sub-Saharan Africa which have attempted to assess prevalence of drug use in pregnancy and its relation to pregnancy outcome [7, 8]. A study in Mozambique reported that antibiotics agents were the most common drugs used (41%), followed by antimalarial drugs (24%). Drug exposure in general was associated with a two fold increase risk of stillbirth [8].

First trimester of pregnancy is the most harmful period for teratogenic exposure because it is when organogenesis takes place albeit, some teratogens may have effect in later stage of pregnancy and may even cause miscarriage [9, 10]. Common medicines such as tetracycline, metronidazole, albendazole, mebendazole, efavirenz (EFV), sulphadoxine-pyrimethamine (SP) and artemisinin-based combination therapy (ACT) are some of therapeutic drugs which are not recommended during first trimester due to fear of embryo-toxicity [11, 12]. All these reported teratogenic drugs and many other which are known or not yet confirmed to have deleterious effects on the foetus are still used by women of childbearing age and pregnant women to treat different illnesses [13, 14]. Thus, there are insufficient safety studies in pregnancy on most drugs used for the treatment of tropical diseases [15].

Demographic Surveillance System (DSS) is an ideal platform to establish pharmacovigilance system in pregnancy. People in DSS area are routinely being followed to update their information in the database. It is therefore easy to identify early enough vital events such as pregnancy, birth and death. A link between the DSS members and health care at the nearby facility can be established with facilitated follow-up of pregnant women. The present study aimed at demonstrating the feasibility of using Health and Demographic Surveillance System (HDSS) as a platform level to monitor drug safety in pregnancy.

## Methods

### Study site and HDSS platform

The study was conducted using the platform of the Rufiji Health and Demographic Surveillance System (HDSS) which is located in Coastal region, Eastern Tanzania. The area has hot weather throughout the year and two rainy seasons. Rufiji HDSS monitors a population of about 97,000; they are all recorded in the database including social and health characteristics. Data from all 13 health facilities within DSS catchment area are also routinely being collected. These health facilities have Reproductive and Child Health (RCH) clinic services. The prevalence of women delivering in health facilities in the study area is 74%. Fertility rate is 4.8 and the maternal mortality ratio is 70 per 100,000 live births [16].

The prevalence of malaria parasitaemia is 14%, and *Plasmodium falciparum* is the predominant species [17]. Malaria is the leading cause of mortality in the district across all ages. It is followed by HIV disease, tuberculosis and pneumonia [18]. Details of the study area and population have been described elsewhere [19].

### Study design and population

#### Enrollment

This was an observational prospective study conducted between April 2012 and March 2013. Pregnant women with a gestational age below 20 weeks and residing in the HDSS area were enrolled in the study and followed until delivery. Participants were recruited from both RCH clinics during their routine clinic visits and from the community through monthly round-based house visits. The set-up of HDSS facilitated early identification of pregnancy status in women of childbearing age through routine HDSS quarterly surveys. A rapid urine pregnancy test was performed for women who were not sure of their pregnancy status.

#### Follow up and pregnant outcome ascertainment

All enrolled participants were followed up on a monthly basis until delivery. Nurses and field workers were responsible for follow-ups in the RCH clinic and community, respectively. Participants were given a unique identification number which was attached to their RCH card and patient’s medical log. These procedures allowed to easily identifying study participants. During enrolment and follow-ups, participants were always encouraged to attend monthly RCH clinic visits and to deliver in the health facility. Women were closely followed at home within two weeks of the expected date of delivery, especially for participant with missing information from the health facility. This allowed to knowing whether the participant had already delivered or not. Even for women delivering at home, they were always encouraged to report to the RCH clinic afterwards. No incentive was given to participants while attending RCH clinic or delivering in the health facility.

A structured questionnaire was used to interview for socio-demographic information, obstetrics and medical history. Physical examination, blood screening test for HIV, syphilis and haemoglobin were performed in the health facility. Patient’s information from RCH card or medical registry was also used for addition information and or clarifying issues.

#### Drug exposure and illness ascertainment

Participants were interviewed for any drug which was taken prior to the enrolment but during the current pregnancy. On the day of enrolment, all women were given a small exercise book as patient’s medical log. The latter was used whenever the woman went to the health facility for treatment or to drug vender to fetch medication. Hence, all clinical information including drug used was filled in this personal medical log. During each monthly follow-up visit, participants were asked for any new drug used, and in all cases evidence for the new used medication was verified from prescription sheet, RCH card, hospital registry or personal medical log. However, in case of discordance between what had been documented in RCH card or medical log and what the participant had reported regarding the used medicine, we relied on participant’s information after further interview to verify specifications of the said medicine. Note that use of a small exercise book as patient’s medical log is routine practice in most of the dispensaries, health centres, and some district hospitals in the country, may be due to challenges of patients’ recordkeeping in most health facilities. Hence, all patients are required by the facility to have their own medical log whereby signs and symptoms of illness, diagnostic findings, and prescriptions are documented by health personnel. Patient’s medical log is kept by the patient and he/she needs to go with it to the health facility whenever seeking for medical attention.

#### Gestational age and newborn assessment

The study recorded the following pregnancy information: pregnancy outcomes (miscarriage, stillbirth or live birth), mother’s complications at delivery, number of babies born, birth weight, gestational age at delivery (estimated from the last date of normal menstrual period [LNMP], or fundal height examination, when LNMP was unknown), and any congenital abnormalities. In the case of home delivery, women were advised to take the baby to the hospital within seven days post-delivery for proper examination.

Congenital abnormalities were assessed post-delivery by a study clinician or health facility midwife. Screening for congenital abnormalities was performed under the guidance of a specifically developed checklist. The screening was limited to identify external abnormalities regardless the degree of severity. No examination was performed to determine neurological scores for sensory or motor patterns. Cases with suspected anomalies were referred to the district and regional hospital for appropriate management.

Pregnancy risk of a drug exposure during pregnancy period was categorized in accordance to US Food and Drug Administration (FDA). US FDA classifies drugs into five categories to describe their risk of teratogenicity; category A (adequate and well-controlled studies have failed to demonstrate a risk to the fetus in all trimesters), category B (animal reproductive studies have failed to demonstrate a risk to the fetus and there are no adequate and well-controlled studies in pregnant women), category C (animal reproductive studies have shown an adverse effect on the fetus and there are no adequate and well-controlled studies in humans, but potential benefits may warrant use of the drug in pregnant women despite potential risks), category D (there is positive evidence of human fetal risk based on adverse reaction data from investigational or marketing experience or studies in human, but potential benefits may warrant use of the drug in pregnant women despite potential risks), and category X (studies in animals or humans have demonstrated fetal abnormalities and/or there is positive evidence of human fetal risk based on adverse reaction data from investigational or marketing experience, and the risks involved in use of the drug in pregnant women clearly outweigh potential benefits) [20].

### Sample size

The sample size was pre-determined by the size of HDSS and the logistically feasible time frame of one year. The number of women in their early pregnancy which could be enrolled was estimated before (as 1000) to be sufficient for pilot implementation of pharmacovigilance system in pregnancy but no formal sample size calculation was performed.

### Primary endpoints

The primary endpoint of the study was pregnancy outcome. Pregnancy outcome included miscarriage, stillbirth or live birth, birth weight and prematurity status at birth. Miscarriage was defined as loss of pregnancy before 28 weeks of gestation, and stillbirth was defined as baby born with no signs of life at or after 28 weeks of gestation. Low birth weight was defined as a birth weight below 2500 g, and premature was defined as birth before 37 weeks of gestational age.

### Statistical analysis

STATA® 12.0 (Stata Corporation, College Station, Texas, USA) was used for data analysis. Numerical variables were summarized into median and range. Categorical variables were summarized using cross tabulation to estimate different proportion. Effects of maternal age, weight and height, gestational age, parity, maternal haemoglobin level, HIV and syphilis status on primary endpoint of the study were assessed by bivariate analysis. Logistic regression models were used to estimate the crude odds ratio (OR) for the association between binary pregnancy outcomes (birth outcome, birth weight and birth maturity status) and medicines exposure. The multivariate adjusted logistic regression model included maternal age and parity as potential confounding variables. Both of the two variables were found to be associated with the study endpoints, having a p value < 0.2 in bivariate analysis, selection criteria to be included in the final model. Two sided Wald test P-values are presented.

### Ethics

Ethical approval for the study was granted by Ifakara Health Institute (IHI) ethical review board and National Institute for Medical Research (NIMR) ethical committee. Written informed consent was obtained from all participants.

## Results

A total of 1089 pregnant women were enrolled into the study and 994 (91.3%) completed the follow-up. The latter constitutes the analysis population. 660 (66.4%) were recruited from the health facility during their routine RCH visits and 334 (33.6%) from the community through house visit. Overall, 323 (32.5%) women were recruited in first trimester of pregnancy with a mean gestational age of 10.4 [standard deviation (SD) 2.3] weeks and 671 (67.5%) in first half of second trimester of pregnancy with mean gestational age of 16.9 (SD 1.7) weeks. Mean gestational age (SD) for participants recruited in the community was 14.2 (4.1) weeks and 15.0 (3.2) for participants recruited in health facilities. Important demographic and clinical characteristics are shown in Table 1.

**Table 1:** **Demographic and clinical characteristics of study women at the time of enrollment (n = 994)**

Characteristics	First trimester	Second trimester	Total
	n = 323	n = 671	n = 994
Mean age, (years)*	26.4 (7.3; 14–49)	26.8 (7.0; 14–46)	26.6 (7.0; 14–49)
Mean BMI*	23.1 (3.8; 14.2-39.6)	23.4 (3.4; 14.0-42.5)	23.3 (3.6; 14.0-42.5)
Mean gestational age, (weeks)*	10.0 (2.2; 3–12)	16.6 (1.9; 13–20)	14.8 (3.6; 3–20)
Gravidity^#^			
Primigravidae	82 (25.4)	198 (29.5)	280 (28.2)
Secundigravidae	62 (19.2)	113 (16.8)	175 (17.6)
3 – 4 pregnancies	99 (30.7)	192 (28.6)	291 (29.3)
≥ 5 pregnancies	80 (24.8)	168 (25.1)	248 (24.9)
Recruited sites^#^			
Health facility	193 (59.8)	467 (69.6)	660 (66.4)
Home	130 (40.2)	204 (30.4)	334 (33.6)
Drinking alcohol^#^	3 (0.9)	3 (0.4)	6 (0.6)
Smoking cigarette^#^	2 (0.6)	0 (0)	2 (0.2)
Mean haemoglobin level, (g/dl)*	7.8 (4.7; 6.0-12.7)	7.5 (4.6; 5.2-14.3)	7.7 (4.6; 5.2-14.3)
HIV status^#^			
Negative	284 (88.0)	603 (89.9)	887 (89.2)
Positive	12 (3.7)	35 (5.2)	47 (4.7)
No results	27 (8.3)	33 (4.9)	60 (6.0)
Syphilis test^#^			
Negative	288 (89.2)	628 (93.6)	916 (92.2)
Positive	9 (2.8)	12 (1.8)	21 (2.1)
No results	26 (8.0)	31 (4.6)	57 (5.7)

*represents data presented in mean, (standard deviation [SD]; range).

^#^represents data presented in number (%).

*Abbreviation*: *BMI* Body Mass Index.

### Episodes of reported illnesses during pregnancy

Out of all enrolled pregnant women, 297 (29.9%) reported to have at least one episode of illness during pregnancy. Six diseases were reported: malaria 14.9% (148), urinary tract infection (UTI) 9.2% (91), sexually transmitted infections (STIs) 3.2% (32), upper respiratory tract infection (URTI) 1.5% (15), diarrhoea 1% (10) and chickenpox 0.1% (1).

### Drugs exposure during pregnancy

15 (1.5%) of all study participants reported not to have used any drug during the pregnancy period. 974 (98%) used any of the three drug groups that are recommended by the Ministry of Health [21] for antenatal intervention, all of which used during second and third trimester of pregnancy. 931 (93.7) used vitamins and mineral supplements. 929 (93.5%) used anthelmintic (mebendazole). 946 (95.2%) used at least one dose of sulfadoxine-pyrimethamine (SP) for intermittent preventive treatment of malaria (IPTp) [735 (73.9%) two doses and 211 (21.2%) one dose] [see Tables 2 and 3].

**Table 2:** **Classes of drugs reported to be used by the pregnant women**

Class of drugs	Number of women exposed (%)
Vitamins and minerals	931 (93.7)
Anthelmintics^α^	929 (93.5)
Analgesics	237 (23.8)
Antibiotics	170 (17.1)
Antimalarials ^α^ *	148 (14.9)
Antifungals	59 (5.9)
Antiretrovirals	29 (2.9)
Traditional medicine	27 (2.7)
Antihistamines	15 (1.5)
Antitussive	8 (0.8)
Antihypertensives	6 (0.6)
Antiasthmatics	5 (0.5)
**Pregnancy risk categories** ^**#**^	
A	931 (93.6)
B	253 (25.5)
C	233 (23.4)
D	46 (4.6)
X	0 (0.0)

^α^See Table 3 for further details.

*Excluding SP for IPTp.

^#^Based on US FDA pregnancy risk categorization.

**Table 3:** **RCH provided drugs and antimalarials exposure in pregnant women (n = 994)**

Drug group	n (%)
SP for IPTp	
Single dose	211 (21.2)
Two doses	735 (73.9)
Not at all	48 (4.8)
Anthelminthic (Mebendazole)	
Yes	929 (93.5)
No	65 (6.5)
Iron and Folic acid supplementation	
Yes	93 (93.7)
No	63 (6.3)
Patients treated for malaria at least once*	
Yes	148 (14.9)
No	846 (85.1)
Types of antimalarials used in treating malaria	
AL only	94 (9.5)
Quinine only	28 (2.8)
SP only	11 (1.1)
AL and Quinine	11 (1.1)
AL and SP	4 (0.4)

*Some women were treated for malaria more than one time during pregnancy period.

*Abbreviation*: *SP* Sulfadoxine-pyrimethamine, *IPPTp* Intermittent Preventive Treatment for malaria in pregnancy, *AL* Artemether-lumefantrine.

For anti-infective drugs used because of illnesses, 170 (17.1%) women used antibiotics, 148 (14.9%) antimalarial drugs, 59 (5.9%) antifungals and 29 (2.9%) antiretrovirals. Some women used more than one type of either of the mentioned anti-infective drugs during their pregnancy period. Tables 2 and 3 summarize drugs exposures during pregnancy among study women.

Based on United State Food and Drug Administration (US FDA) risk categorization of drugs in pregnancy, the most common drugs used under category ‘A’ were ferrous sulfate and folic acid, category ‘B’ paracetamol, amoxicillin, erythromycin, metronidazole, benzathine benzylpenicillin and ceftriaxone, category ‘C’ antimalarial for treating illness (AL, quinine, SP), antiretroviral (ARV) for HIV infection (zidovudine, lamivudine and nevirapine), doxycycline, cotrimoxazole, aspirin, diclophenac, hyoscine butylbromide and promethazine, category ‘D’ traditional medicines and phenobarbitone, and no category ‘X’.

### Pregnancy outcome

Out of 994, 897 (90.2%) women delivered in health facilities, 94 (9.5%) at home, and 3 (0.3%) along the road side on their way to the health facility. There were three maternal deaths which all occur within 24 hours post-delivery, two of them due to post-partum haemorrhage and one secondary to eclampsia. Pregnancy outcomes included 28 (2.8%) abortions, 41 (4.1%) stillbirth and 925 (93.1%) live births. Regarding birth outcomes, 99 (10.0%) were premature and 55 (5.0%) babies had low birth weight. 12 (1.2%) of the newborns were identified as having congenital anomalies at the time of birth: 8 were polydactyl and the remaining 4 had clubfoot, spina bifida, genital defect or cardiac defect. Two women with a newborn having polydactyl each were exposed to ARV and antitussive (coughing syrup), respectively and both drugs are under US FDA risk category ‘C’. One woman with a newborn having *spina bifida* was exposed to phenobarbitone in third trimester, the drug which is in US FDA risk category ‘D’. The remaining women with congenital anomalies babies were not exposed to neither US FDA category ‘C’ nor category ‘D’ drugs.

### Relation of medication exposure to pregnancy outcome

Maternal age and parity were assessed to determine their effect on pregnancy outcome (as potential confounders of drug effect). Maternal age had no significant effect on birth weight (0R 1.0; p value 0.356) but was associated with 3% increased risk of premature birth (OR 1.03; p value 0.038) and 4% increased risk of miscarriage/stillbirth (OR 1.04; p value 0.020). Parity had no significant effect on miscarriage/stillbirth (OR 1.1; p value 0.690) but was associated with a 60% increase risk of preterm birth (OR 1.6; p value 0.065) and 60% decreased risk of low birth weight (OR 0.6; p value 0.116).

With the level of risk assessment that this study was powered, antimalarial exposure during pregnancy was not significantly associated with an increased risk of miscarriage/stillbirth (adjusted OR 1.3; 95%CI 0.7 -2.4; p = 0.494), low birth weight (adjusted OR 0.7; 95CI% 0.3 – 1.8; p = 0.460) or premature birth (adjusted OR 1.2; 95%CI 0.6 – 2.7; p = 0.629). Antibiotics exposure was neither associated with an increased risk of miscarriage/stillbirth (adjusted OR 0.8; 95% CI 0.4 – 1.6; p = 0.526), low birth weight (adjusted OR 0.6; 95% CI 0.2 – 1.6; p = 0.295) or premature birth (adjusted OR 1.4; 95% CI 0.7 – 2.8; p = 0.348) [Table 4].

**Table 4:** **Antimalarial and antibiotics exposure in relation to pregnancy outcome (n = 994)**

Variables	Outcomes		Crude OR	P ^μ^	Adjusted OR ^α^	P ^μ^
			(95% CI)		(95% CI)	
**Birth outcome**	**MC/SB**	**Live birth**				
	**n (%)**	**n (%)**				
Antimalarial exposure*			1.2 (0.6 – 2.3)	0.546	1.3 (0.7 – 2.4)	0.494
Yes	12 (17.4)	136 (14.7)				
No	57 (82.6)	789 (85.3)				
Antibiotics exposure			0.8 (0.4 – 1.6)	0.551	0.8 (0.4 – 1.6)	0.526
Yes	10 (14.5)	160 (17.3)				
No	59 (85.5)	765 (82.7)				
**Birth weight (grams)**	**< 2500**	**≥ 2500**				
	**n (%)**	**n (%)**				
Antimalarial exposure*			0.7 (0.3 – 1.9)	0.523	0.7 (0.3 – 1.8)	0.460
Yes	5 (11.4)	131 (14.9)				
No	39 (88.6)	750 (85.1)				
Antibiotics exposure			0.6 (0.2 – 1.5)	0.291	0.6 (0.2 – 1.6)	0.295
Yes	5 (11.4)	155 (17.6)				
No	39 (88.6)	726 (82.4)				
**Maturity status at birth**	**Preterm**	**Term**				
	**n (%)**	**n (%)**				
Antimalarial exposure*			1.1 (0.5 – 2.5)	0.742	1.2 (0.6 – 2.7)	0.629
Yes	8 (16.3)	128 (14.6)				
No	41 (83.7)	748 (85.4)				
Antibiotics exposure			1.4 (0.7 – 2.8)	0.329	1.4 (0.7 – 2.8)	0.348
Yes	11 (22.5)	149 (17.0)				
No	38 (77.5)	727 (83.0)				

MC/SB = Miscarriage or stillbirth; OR = odds ratio; CI = confidence interval.

*Excluding SP for IPTp.

^μ^Estimated from the logistic regression model with Wald type P-value.

^α^Adjusted for parity and maternal age.

Exposure to drugs under US FDA pregnancy risk category ‘A’, which mainly included ferrous sulfate and folic acid were associated with a reduced risk of miscarriage/stillbirth (adjusted OR 0.1; 95% CI 0.08 – 0.3; p < 0.001). There was no significant association of adverse pregnancy outcome in relation to exposure to drugs under category ‘B’, ‘C’ and ‘D’ [Table 5].

**Table 5:** **US FDA pregnancy risk categories of drugs exposure in relation to pregnancy outcome**

Variables	Outcomes		Crude OR	P ^μ^	Adjusted OR ^α^	P ^μ^
			(95% CI)		(95% CI)	
**Birth outcome**	**MC/SB**	**Live birth**				
	**n (%)**	**n (%)**				
Drugs category ‘A’			0.1 (0.1 – 0.3)	< 0.001	0.1 (0.08 – 0.3)	< 0.001
Yes	51 (73.9)	880 (95.1)				
No	18 (26.1)	45 (4.9)				
Drugs category ‘B’			0.6 (0.3 – 1.1)	0.115	0.6 (0.3 – 1.1)	0.111
Yes	12 (17.4)	241 (26.1)				
No	57 (82.6)	684 (73.9)				
Drugs category ‘C’			1.4 (0.8 – 2.3)	0.261	1.4 (0.8 – 2.4)	0.257
Yes	20 (29.0)	213 (23.0)				
No	49 (71.0)	712 (77.0)				
Drugs category ‘D’			1.0 (0.3 – 2.9)	0.974	0.9 (0.3 – 3.2)	0.939
Yes	3 (4.3)	41 (4.4)				
No	66 (95.7)	884 (95.6)				
**Birth weight (grams)**	**< 2500**	**≥ 2500**				
	**n (%)**	**n (%)**				
Drugs category ‘A’			0.5 (0.2 – 1.4)	0.191	0.5 (0.2 – 1.5)	0.207
Yes	40 (90.9)	840 (95.4)				
No	4 (9.1)	41 (4.6)				
Drugs category ‘B’			0.5 (0.2 – 1.2)	0.122	0.5 (0.2 – 1.2)	0.125
Yes	7 (15.9)	234 (26.6)				
No	37 (84.1)	647 (73.4)				
Drugs category ‘C’			0.7 (0.3 – 1.6)	0.436	0.7 (0.3 – 1.6)	0.387
Yes	8 (18.2)	205 (23.3)				
No	36 (81.2)	676 (76.3)				
Drugs category ‘D’			0.5 (0.1 – 3.6)	0.485	0.6 (0.1 – 3.4)	0.449
Yes	1 (2.3)	40 (4.5)				
No	43 (97.7)	841 (95.5)				
**Maturity status at birth**	**Preterm**	**Term**				
	**n (%)**	**n (%)**				
Drugs category ‘A’			0.6 (0.2 – 1.6)	0.277	0.5 (0.2 – 1.5)	0.231
Yes	45 (91.8)	835 (95.3)				
No	4 (8.2)	41 (4.7)				
Drugs category ‘B’			1.5 (0.8 – 2.8)	0.160	1.5 (0.8 – 2.8)	0.175
Yes	17 (34.7)	224 (25.6)				
No	32 (65.3)	652 (74.4)				
Drugs category ‘C’			1.4 (0.7 – 2.6)	0.345	1.4 (0.8 – 2.7)	0.269
Yes	14 (28.6)	199 (22.7)				
No	35 (71.4)	677 (77.3)				
Drugs category ‘D’			2.0 (0.7 – 5.9)	0.201	2.0 (0.7 – 6.0)	0.200
Yes	4 (8.2)	37 (4.2)				
No	45 (91.8)	839 (95.8)				

MC/SB = Miscarriage or stillbirth; OR = odds ratio; CI = confidence interval.

^μ^Estimated from the logistic regression model with Wald type P-value.

^α^Adjusted for parity and maternal age.

## Discussion

The present study shows that there is a considerable amount and several types of drugs exposure during pregnancy in this region, as it may apply to other parts of Tanzania and sub Saharan countries. To our knowledge, it is the first prospective study conducted in a resource-limited setting that attempted to demonstrate the feasibility of establishing a reliable pregnancy exposure registry which followed a large group of pregnant women from their early pregnancy stage [7, 22]. All drugs exposure and related diseases during pregnancy period were carefully identified and recorded.

More than 98% of study women reported to have used at least one medication during pregnancy. This is more than twice to what was observed in Mozambique in a study conducted seven years ago [8]. Most of the drugs used were the ones covered under antenatal intervention program. The coverage of anthelmintic, haematemic and SP for IPTp in our study was almost twice that estimated at national level [23]. This high use of drugs may be the result of intense health promotion activities in the area under HDSS, in close collaboration with local and government authorities. A 94% coverage for iron and folic acid supplementations in this rural area is a remarkable achievement. Apart from haematemic, anthelminthic and IPTp-SP exposure, analgesics were the most reported prescribed drugs. This observation is in agreement with two previous studies in sub-Saharan Africa [8, 24]. Over- or under- reporting of drug use is of course possible due to recall bias, particularly for women enrolled in an advanced gestational age and with poor documentation of the medicine used. Errors on gestational age measurement cannot be excluded as well.

Malaria was the most often recorded illness during pregnancy (15%). This illustrates the high intensity of transmission in the study area [16] and the vulnerability of pregnant women to malaria [25]. It highlights the importance of having safe and effective drugs to clear parasites during pregnancy. AL was prescribed nearly 3 times more often than quinine. Some of these treatments correspond to inadvertent exposure, similarly to what has been observed in Sudan and Zambia [26, 27]. Others represent treatments that were probably administered during second and third trimester, as recommended [12]. A better availability of AL when compared to quinine in health facilities and drug shops [2] may have also contributed to the frequent use of this drug.

When taken as a category and irrespective of the timing during pregnancy, antimalarial and antibiotic exposures were not associated with adverse pregnancy outcome. This result should be interpreted with caution since different types of medications, or the same medication but given at different time during pregnancy, may have different effects. A more detailed assessment of antimalarial exposure, taking into account the type and time of exposure during pregnancy have been reported elsewhere in a complementary study which included a larger sample size of pregnant women from two HDSS areas [14]. The present paper was more to pilot the feasibility of a pharmacovigilance system embedded in a HDSS in a developing country.

Iron and folic acid supplementation, the main drugs under US FDA pregnancy risk category ‘A’ were protective against miscarriage/stillbirth. However, adherence to these supplements and number of doses prescribed were not assessed. There was no much evidence yet to support the added benefits of these supplements in preventing miscarriage or stillbirth. Evidence mainly supports the use of these drugs to prevent anaemia and iron deficiency at term, to reduce the risk of low birth weight and early neonatal death, all factors that have shown to have a beneficial impact on child’s survival [28–30]. The observed beneficial effects of recommended iron and folic acid supplementation in pregnancy validate the concept of pharmacovigilance system through HDSS.

About 3% of study women used traditional medicines which in most cases are under pregnancy risk category ‘D’. The use of traditional medicines may have been higher than what is reported in the present study since participants were interviewed by health care providers who are trained to discourage patients to use herbs. Underreporting is a well-known phenomenon in other developing countries whereby study participants had difficulties to disclose use of traditional medicine to the health care professionals [8, 31].

The observed prevalence of 1.2% congenital anomalies in the study is lower compared to the 3.0% global prevalence estimated by the WHO [32]. There is no national register to compare our rate with that in other parts of the country. The observed rate of congenital anomalies in the present study may be underestimated because screening for anomalies was limited to the external ones and was carried out only once, at the time of delivery. Hence, there are possibilities of more anomalies to be identified later in life as the child grows. It would be important to follow all delivered babies prospectively at defined intervals, at least until the age of one year to monitor sensory and motor developmental milestones. Such a monitoring can be easily implemented in HDSS settings. In addition, it is also important to consider improving newborn’s screening standards, training of health staff and detailed birth registry records to implement a reliable pregnancy pharmacovigilance system [22].

The present study demonstrates a way forward to establish a feasible, reliable and manageable active pharmacovigilance system in a resource-limited setting by taking advantage of existing monitoring platforms such as HDSS. Pregnancy pharmacovigilance system under HDSS platform may be cost-effective due to the already existing infrastructure such as personnel and established database constantly updated. Feasibility of the present proposed pharmacovigilance system is of merit over the probabilistic record linkage for monitoring antimalarial safety evaluated in Senegal [7] which is subjected to bias because of poor medical record system in most health facilities in developing countries and hence, some exposure cases and pregnancy outcome information may easily be missed. The drug exposure pregnancy registry proposed by Mehta U *et al*. [22] appears to be promising but its operational costs may be very high in most resource-limited countries where presence of skilled medical personnel is still a big problem.

Pharmacovigilance systems should reduce the uncertainty about safety of newly marketed medication against tropical diseases. Such a system that collects systematically reliably data to determine whether a given medication is teratogenic or not through monitoring of a large exposure group could provide strong evidence on safety [13, 22]. It could help to overcome the current shortfall which is commonly seen in medical practice when treating a pregnant woman with medication that has suboptimal efficacy because of potential safety problems. Also, it could assist in the assessment of medicines that are not recommended during pregnancy, but are sometimes not avoidable to save the mother or the unborn child.

## Conclusion

Almost all women are exposed to medication during pregnancy, either because drugs are recommended during this period, or because women are sick and need treatment. Since exposure to contraindicated drugs during pregnancy is sometimes inevitable in either trimester, safety monitoring mechanism should be in place in order to generate reliable information for the promotion of safe and effective treatment during pregnancy. HDSS sites can have a useful role in providing reliable pharmacovigilance data and the experience from its success will be helpful to expand the system to none HDSS areas.
